# Transcending Lifshitz Theory: Reliable Prediction of Adhesion Forces between Hydrocarbon Surfaces in Condensed Phases Using Molecular Contact Thermodynamics

**DOI:** 10.1021/acs.langmuir.3c03218

**Published:** 2024-06-27

**Authors:** Oscar Siles Brügge, Christopher A. Hunter, Graham J. Leggett

**Affiliations:** † Department of Chemistry, University of Sheffield, Brook Hill, Sheffield S3 7HF, U.K.; ‡ Department of Chemistry, University of Cambridge, Lensfield Road, Cambridge CB2 1EW, U.K.

## Abstract

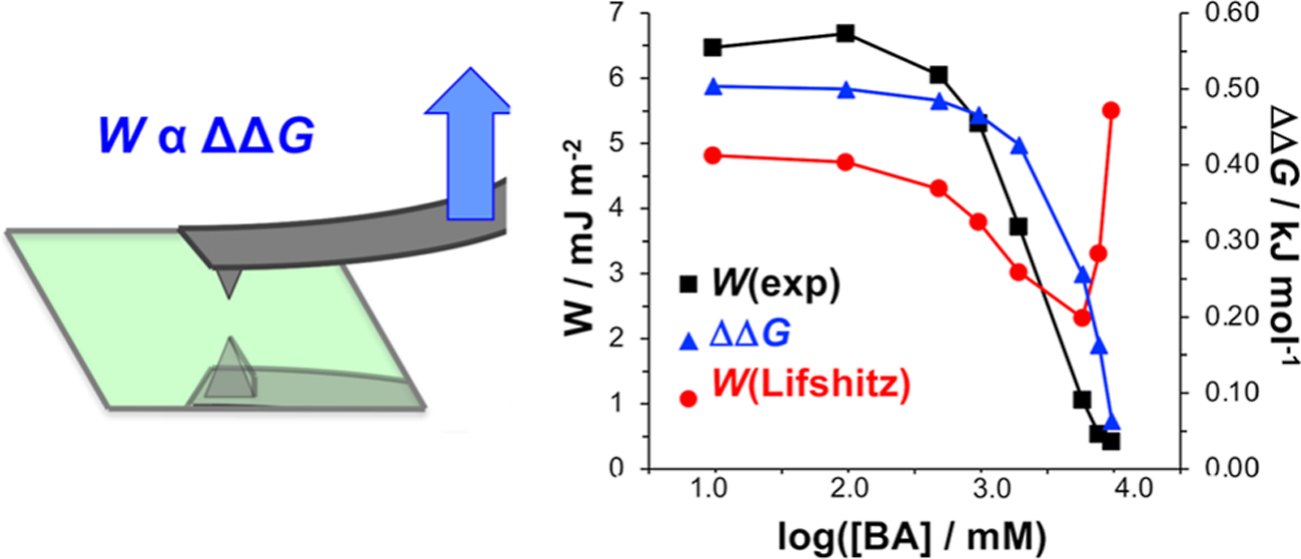

Lifshitz theory is widely used to calculate interfacial interaction energies and underpins established approaches to the interpretation of measurement data from experimental methods including the surface forces apparatus and the atomic force microscope. However, a significant limitation of Lifshitz theory is that it uses the bulk dielectric properties of the medium to predict the work of adhesion. Here, we demonstrate that a different approach, in which the interactions between molecules at surfaces and in the medium are described by a set of surface site interaction points (SSIPs), yields interaction free energies that are correlated better with experimentally determined values. The work of adhesion *W*(Lifshitz) between hydrocarbon surfaces was calculated in 260 liquids using Lifshitz theory and compared with interaction free energies ΔΔ*G* calculated using the SSIP model. The predictions of these models diverge in significant ways. In particular, ΔΔ*G* values for hydrocarbon surfaces are typically small and vary little, but in contrast, *W*(Lifshitz) values span 4 orders of magnitude. Moreover, the SSIP model yields significantly different ΔΔ*G* values in some liquids for which Lifshitz theory predicts similar values of *W*(Lifshitz). These divergent predictions were tested using atomic force microscopy. Experimentally determined works of adhesion were closer to the values predicted using the SSIP model than Lifshitz theory. In mixtures of methanol and benzyl alcohol, even greater differences were found in the interaction energies calculated using the two models: the value of ΔΔ*G* calculated using the SSIP model declines smoothly as the benzyl alcohol concentration increases, and values are well correlated with experimental data; however, *W*(Lifshitz) decreases to a minimum and then increases, reaching a larger value for benzyl alcohol than for methanol. We conclude that the SSIP model provides more reliable estimates of the work of adhesion than Lifshitz theory.

## Introduction

Interfacial interactions regulate a multitude of phenomena,^1^ including adhesion,^2,3^ wetting,^4−7^ friction,^8^ and biological processes^9^ such as protein adsorption,^10,11^ tissue cell attachment,^10,12−14^ and biofilm formation.^15,16^ Many techniques have been developed to measure adhesive interactions at surfaces, including the surface forces apparatus,^17−19^ atomic force microscope,^11,20−27^ contact angle measurement,^1,28^ peel tests,^29^ and others. To calculate adhesive energies from such measurements, a quantitative model is required that relates the observables (e.g., forces) to the physical properties of the interacting materials (e.g., interfacial free energies). There are well-established models for the van der Waals attractive forces between molecules in vacuum, and in the gas phase, pairwise additivity of forces is usually assumed. However, in condensed phases, the assumption of pairwise additivity breaks down.^1^ The Lifshitz model solves this problem by treating interacting media as continuous phases and using the mean bulk dielectric properties of interacting phases to calculate the van der Waals forces.

The foundations of the Lifshitz model lie in quantum field theory,^30,31^ but subsequently a number of simplifications have been made to broaden its applicability, notably the modifications due to Israelachvilli.^1^ Lifshitz theory is used to calculate the Hamaker constant *A* which, together with other terms describing the interacting system, may be used to calculate adhesive energies at interfaces.^27^ For example, the interaction energy *W* is given by *W* = −*AR*/6*D* for a hemisphere of radius *R* interacting with a planar counter surface at a distance *D*.^1^ This equation provides a realistic model for an atomic force microscopy (AFM) probe and also for two crossed cylinders with equal radii *R*, as in the surface forces apparatus.^1^

Although the Lifshitz theory is well-established, the use of mean bulk dielectric properties to predict interaction energies between surfaces in condensed phases seems intuitively to be problematic. Treating the interacting surfaces as slabs neglects the heterogeneity of molecular interfaces. Moreover, adhesive interactions are thermodynamically irreversible, leading to hysteresis in experimental measurements of interactions at surfaces, and the description of such phenomena requires an approach that is grounded in thermodynamics. Other important phenomena, such as the hydrophobic effect, are also thought to have their origins in interfacial thermodynamics. Thus, a general approach to modeling interfacial adhesive energies that is rooted in the thermodynamics would appear to offer significant advantages.

In this paper, we use a molecular-scale thermodynamic model for noncovalent interactions in liquids and the surface site interaction point (SSIP) model^32−36^ to predict interaction free energies between hydrocarbon surfaces in 260 different liquids. In the SSIP model, noncovalent interaction free energies are determined via the attribution of local interaction parameters to specific sites on molecular surfaces.^32,36^ These interaction parameters are determined either experimentally or theoretically, and they may be used to calculate the interaction energy and its dependence on the medium. We contrast the predictions of the SSIP model, based on a molecular treatment of functional group interactions between the interacting surfaces and the medium, with those of the Lifshitz model,^30,31,37,38^ in which the interacting surfaces are treated as slabs and the van der Waals force is determined by the mean dielectric properties of the media.^1^

In earlier work, we found that the SSIP model yielded predictions for carboxylic acid-terminated surfaces that diverged from those of the Lifshitz model.^39^ However, it might be argued that this is expected for surfaces containing permanent dipoles that can form directional hydrogen bonds.^40^ In contrast, hydrocarbon surfaces might be expected to behave in greater conformity with the predictions of Lifshitz theory because their interactions are dominated by polarization forces.^1,27^ However, we demonstrate here that for hydrocarbon surfaces, significant differences are found between interaction energies calculated using Lifshitz theory and the SSIP model. The experimental works of adhesion obtained using atomic force microscopy are predicted more accurately by the SSIP model than by Lifshitz theory.

Several groups have sought previously to test the predictions of Lifshitz theory using force measurements made using AFM. Of particular importance is the work of Spencer and coworkers, who calculated work of adhesion values for AFM probes interacting with polymer films using Lifshitz theory.^27^ They compared measurements made in water, isopropanol, and perfluorodecalin and found that the adhesion force was correlated with the calculated work of adhesion. Building on this, previous work in the authors’ laboratory indicated that for hydrocarbon surfaces, pull-off forces measured by AFM were correlated with works of adhesion calculated using Lifshitz theory in liquids that did not act as hydrogen-bond donors.^41^ However, a limited range of liquids were used in this earlier work.

In the present paper, we describe the results of a much more extensive investigation, involving calculations of interaction energies in 260 liquids with widely varying properties. We calculated the work of adhesion *W*(Lifshitz) using Lifshitz theory and compared these values with interaction free energies ΔΔ*G* determined using the SSIP model. While for some liquids the two models yield similar values for the work of adhesion, in other liquids, the predictions are divergent. These predictions were tested using AFM by applying well-established contact mechanics treatments^27,39,42,43^ to determine the experimental work of adhesion. Our data indicate that where the models diverge, it is the predictions of the SSIP model that are the most closely correlated with the experimental data.

## Methods

### Determination of the Work of Adhesion Using the Lifshitz Model

The work of adhesion between identical nonpolar surfaces (1) in a liquid medium (3) according to Lifshitz theory was calculated by first determining the Hamaker constant of the system^1^
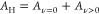
1where the Hamaker constant (*A*_H_) is the sum of a zero-frequency term *A*_*n*=0_ due to Keesom (dipole–dipole) and Debye (induced-permanent dipole) interactions and a nonzero frequency term *A*_*n*>0_ arising from long-range London dispersion forces. Both of these terms were calculated using the approximate equation derived by Israelachvili.^1^ The zero-frequency term is a function of the dielectric constants of the surfaces and liquid medium (*e*_1_ and *e*_3_, respectively) as^1,27^
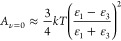
2where *k* is the Boltzmann constant (1.38065 × 10^–23^ J K^–1^) and *T* is the absolute temperature (assumed 298.15 K). The nonzero frequency term, determined by the refractive indices of the surfaces and liquid medium (*n*_1_ and *n*_3_, respectively), is^1,27^
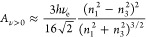
3where *h* is the Planck constant (6.62608 × 10^–34^ J s) and *n*_e_ the main electronic absorption frequency in the UV (assumed 3 × 10^–15^ s^–1^ for all media). For binary mixtures of methanol and benzyl alcohol, experimentally obtained refractive indices were used, and a linear change in dielectric constants was assumed. From the Hamaker constant, the work of adhesion was calculated using the relation^1^
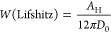
4where *D*_0_ the closest separation between the two surfaces. A value of *D*_0_ = 0.165 nm has been shown to provide reasonable estimates of the surface energies of various organic materials^1,44^ and was therefore used for this study.

In this work, the hydrocarbon film being investigated is supported on a gold film. It is necessary to consider whether the gold substrate is likely to exert an influence on the measurements. Miller and Abbott used Lifshitz theory to model the influence of van der Waals forces associated with metal substrates on the spreading of liquid drops on SAMs formed by the adsorption of alkylthiols HS(CH_2_)_*n*_CH_3_.^45^ They found that for *n* < 10, the substrate exerted a significant effect on the liquid drop, but that for *n* > 10, the effect of the substrate was small. Nevertheless, we additionally carried out calculations of the work of adhesion using a five-layer model, consisting of two hydrocarbon films (2 and 2′) of thicknesses *T* and *T*′ supported on gold films (1 and 1′) interacting in a medium (3). To facilitate this, a slightly amended calculation is required that takes into account all the different interactions^1^
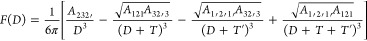
5where *F*(*D*) is the nonretarded van der Waals force at a separation *D*, and all individual Hamaker constants are calculated using the method described above. In order to determine the work of adhesion, the force function was integrated over all separations
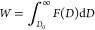
6

The difference in the work of adhesion calculated using the two different approaches was found to be very small (see the Supporting Information), in agreement with expectations based on the work of Miller and Abbott for dodecanethiolate SAMs.^45^ In this work, *W*(Lifshitz) is calculated using eq 1. However, works of adhesion calculated using the five-medium model are additionally shown in Table 1 above for completeness.

**Table 1:** Relative Permittivities (ε), Refractive Indices (*n*_D_, Measured at Sodium D Line), Adhesion Forces *F*_po_ and Works of Adhesion *W*(exp) Determined from AFM Measurements, and Works of Adhesion Calculated Using the Lifshitz [*W*(Lifshitz)] and SSIP Models [*W*(SSIP)] for DDT SAMs Interacting in a Range of Pure Liquids^a^

liquid	*n*_D_	ε	*F*_po_/*R*/mN m^–^^1^	*W*(exp)/mJ m^–^^2^	*W*(Lifshitz)/mJ m^–^^2^	*W*(SSIP)/mJ m^–^^2^
DDT	1.420	2.00				
water	1.333	78.36	292 ± 12	46.5 ± 1.9	4.98 (5.03)	37.08
methanol	1.327	32.66	41 ± 4	6.52 ± 0.53	4.95 (5.03)	7.19
ethanol	1.359	24.55	34 ± 4	5.41 ± 0.63	3.26 (3.37)	5.61
nitromethane	1.379	35.87	30 ± 3	4.77 ± 0.51	2.91 (2.99)	3.80
benzyl alcohol	1.538	12.70	8.3 ± 2.1	1.32 ± 0.33	5.31 (5.47)	3.28
benzonitrile	1.525	25.20	4.2 ± 1.6	0.67 ± 0.25	5.11 (5.22)	2.03
*n*-heptane	1.385	1.92	2.8 ± 1.0	0.45 ± 0.16	0.39 (0.38)	0.72
*n*-decane	1.410	1.99	3.8 ± 1.1	0.60 ± 0.18	0.04 (0.06)	0.73
*n*-dodecane	1.420	2.00	2.4 ± 0.9	0.38 ± 0.14	0.00 (0.00)	0.78
*n*-hexadecane	1.433	2.05	1.7 ± 1.0	0.27 ± 0.16	0.04 (0.06)	0.81
1,2,4-trichloro-benzene	1.571	4.15	2.7 ± 1.4	0.43 ± 0.22	6.36 (6.52)	0.81

a *W*(Lifshitz) data were calculated using eq 1 with five medium data in parentheses. Bulk values for DDT are included for reference. ε, *nD* average values at 20 °C obtained from Marcus et al.^66^ and Lide et al.^67^

### Determination of the Interaction Free Energy ΔΔ*G* Using the SSIP Model

The free energy of complexation for an equivalent system of interacting surfaces may be obtained from the SSIP model introduced by Hunter.^32^ The model extends previous work in estimating the solvent effects on equilibrium constants for solute–solute interactions.^32^ Interactions between a molecule and neighboring molecules in a liquid are described by a set of SSIPs, each of which represents a molecular surface area of 9.5 Å^2^ and a volume of 5 Å^3^. An electrostatic interaction parameter, ε_i_, is obtained for each SSIP from the molecular electrostatic potential surface calculated using density functional theory and used to calculate the polar contribution to the interaction between two SSIPs. The nonpolar contribution to the interaction between two SSIPs, *E*_VDW_ = −5.6 kJ mol^–1^, can be treated as a constant because each SSIP represents the same molecular surface area.^32,46^ The free energy change for the interaction between two solute SSIPs in a liquid is calculated using the SSIMPLE algorithm, which treats the liquid phase as an ensemble of interacting SSIPs at equilibrium. All SSIP interactions are treated in a pairwise manner, such that the association constant for interaction between the *i*th and *j*th SSIP, *K*_*ij*_, is given by
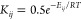
7
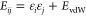
8

Given the total concentration of each SSIP, eqs 7 and 8 can be used to construct a set of simultaneous equations that can be solved to determine the speciation of SSIP contacts in the liquid. The solvation free energy change for solute 1 is determined from the fraction of free SSIPs (1_f_) in the solution
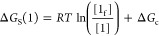
9where *R* is the gas constant (8.31446 J K^–1^ mol^–1^), *T* is the standard temperature (298.15 K), and Δ*G*_c_ is the confinement free energy that is required to describe phase change equilibria, but in the case will cancel out (see eqs 10 and 11).

The free energy change for the binding of solute 1 into a complex with another solute 2 is described in the same way by treating the complex as a pure phase containing only the solute SSIPs at the same total density as the liquid
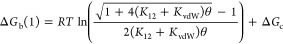
10where *K*_12_ is the association constant for the interaction between solute SSIP 1 and solute SSIP 2 calculated using eqs 6 and 7, *K*_vdW_ is the corresponding association constant for a nonpolar interaction (*E*_*ij*_ = *E*_vdW_), and θ is the total SSIP density of the liquid phase.

The free energy change associated with the exchange of solvent and solute interactions when a complex is formed is given by
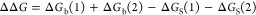
11

To model two interacting nonpolar surfaces, the electrostatic interaction parameters for alkanes were used (ε_1_ = 0.5 and ε_2_ = −0.5) were used to represent the surfaces as two interacting solute SSIPs present at low concentrations relative to the solvent (1 mM). The calculated values of free energy change were then normalized such that a greater value indicates a greater affinity for the surfaces to form a complex.

### Interactions in Liquid Mixtures

While solvent mixtures are natively possible in the SSIP model, for Lifshitz theory, the bulk dielectric constant and refractive index are required. The dielectric constant of a nonpolar mixture can be calculated using the Clausius–Mosotti equation^47^
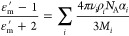
12where ε_*m*_^′^ is the dielectric constant of the mixture, *N*_A_ is Avogadro’s number, and for each component *i* of the mixture *n*_*i*_ is the volume fraction, *r*_*i*_ is the mass density, α_*i*_ is the electric polarizability, and *M*_*i*_ is the molecular weight. The values of these parameters can be obtained from the literature. However, it has been found that the dielectric constants of mixtures with one or two nonpolar liquids typically display a broadly linear relationship with composition. For mixtures containing two polar liquids, a linear relation between the dielectric constant and the composition was found to yield average deviations of up to 5% at 298 K. As such, a linear relation was assumed when determining the dielectric constant of the resulting mixtures. For the refractive index, the Lorentz–Lorenz mixing rule was used as it has been previously shown to yield average deviations of less than 2% for binary systems of mixtures of several types of liquids^48,49^
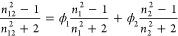
13where *n*_12_ is the refractive index of the mixture, *n*_1_ and *n*_2_ are the refractive indices of the two pure components, and *f*_1_ and *f*_2_ are the volume fractions. For mixtures of benzyl alcohol and methanol, experimentally obtained values for the refractive index of the mixture were used.

## Experimental Section

### Modeling

Due to the need to calculate the interaction between surfaces according to both Lifshitz theory and the Hunter model for a large number of solvents, a custom piece of software named “TToolbox” (short for Tribology Toolbox) was written in C#.^50^ This allowed for input parameters to be easily modified in a graphical user interface (GUI) by the user, greatly accelerating the usual workflow of calculating interfacial properties. Additionally, TToolbox was written following the object-oriented programming (OOP) paradigm, allowing for code to be modular and reusable. As a result of this modularity, TToolbox was expanded to allow for the bulk processing of AFM files, as well as basic statistical analysis of these. MATLAB scripts were originally written for this purpose and form the basis of the algorithms used in TToolbox.

### Monolayer Formation

Self-assembled monolayers (SAMs) of 1-dodecanethiol (DDT, 99%, Sigma-Aldrich) were formed on gold-coated glass substrates using previously published methodology. Glass slides (Menzel-Gläser, 22 × 50 mm, #1.5, Braunschweig, Germany) were first cleaned in piranha solution (a mixture of concentrated sulfuric acid and hydrogen peroxide with a 70:30 volume ratio; Caution! Piranha solution is a strong oxidizing agent and can detonate unexpectedly on contact with organic materials) and rinsed thoroughly in deionized water (Veolia Water Technologies, High Wycombe, UK). The slides were then immersed in RCA solution (H_2_O_2_/NH_3_/H_2_O, 1:2:5 volume ratio) for 30 min and rinsed thoroughly in deionized water after cooling. The slides were allowed to dry for at least 2 h in a clean 120 °C oven before metal evaporation. Gold (Goodfellow Advanced Materials, Cambridge, UK) was deposited at a rate of using an Edwards Auto 306 thermal evaporation system. After deposition of the metal film, the substrates were immersed in a degassed solution of DDT in ethanol [high-performance liquid chromatography (HPLC) grade, Sigma-Aldrich] for 18 h. The samples were rinsed with ethanol and dried under a stream of nitrogen before use. SAMs were characterized carefully using contact angle measurement, X-ray photoelectron spectroscopy, and AFM (further details are given in the Supporting Information). The spectra acquired and the contact angle measurement data were in exact agreement with expectations for a dense, close-packed SAM of dodecanethiolate, based on the very large body of the literature on these very widely studied materials.

### AFM Probe Preparation

Functionalized AFM probes were prepared using established methodology supported by extensive literature.^51−58^ Commercial V-shaped silicon nitride AFM probes (DNP-10, Bruker AFM Probes) with a nominal spring constant of 0.12 N m^–1^ were used for force spectroscopy and friction force measurements. Previous studies have shown that these commercially available AFM probes, usually supplied in protective gel packs, have high levels of polydimethylsiloxane contamination. Due to damage observed in the AFM probes after piranha cleaning (the reflective layer is often damaged), the probes were cleaned using a ProCleaner Plus UV/o cleaner (BioForce, Salt Lake City, USA) for 30 min. After exposure to ozone, the probes were rinsed in HPLC-grade ethanol and gently dried in a stream of N_2_. Probes and slides for DDT SAM formation were coated with a 1 nm chromium (Cr, 99.5%, Sigma-Aldrich) adhesive layer at a rate of 0.01 nm s^–1^, followed by a 10 nm layer of gold (Au, 99.999%, Goodfellow metals) deposited at 0.03 nm s^–1^ in an Edwards Auto 306 thermal evaporator with bell jar and diffusion pump at operating pressures of 10^–6^ mbar.

SAM-functionalized probes were prepared by immersion of gold-coated probes in a 1 mM solution of DDT in degassed HPLC-grade ethanol for 24 h. Probes were washed with copious amounts of HPLC-grade ethanol and dried in a stream of N_2_ before use.

### Atomic Force Microscopy

*n*-Heptane (HPLC, Fisher Scientific), water (18 MΩ), ethanol (HPLC, Fisher Scientific), methanol (anhydrous, 99.8%, Sigma-Aldrich), benzyl alcohol (anhydrous, 99.8%, Sigma-Aldrich), benzonitrile (anhydrous, ≥99%, Sigma-Aldrich), and 1,2,4-trichlorobenzene (anhydrous, ≥99%, Sigma-Aldrich) were all used as received and injected into the AFM fluid cell using a piranha-cleaned glass syringe.

All measurements were made on a NanoScope V MultiMode 8 (Bruker UK Ltd., Coventry, UK) in conjunction with a J-scanner. Calibration of the lateral and normal forces was performed in two stages. The normal spring constant was calibrated at the beginning of all experimental procedures for any given probe using the thermal noise technique first described by Hutter and Bechhoefer,^59^ implemented via the calibration routine in the microscope operating system, with a correction factor of 0.93 for V-shaped AFM probes.

To enable the accurate quantification of lateral forces, the lateral stiffness of every probe was calibrated using the wedge calibration method introduced by Ogletree et al.^60^ and adapted to include adhesion by Varenberg et al.^61^ The work of Ogletree et al. provides a detailed analysis of the mechanical behavior of triangular cantilevers and demonstrates clearly the relationship between the measured deflection of the cantilever and the friction force. To facilitate probe calibration, friction measurements across a flat and inclined surface were required. A commercially available silicon calibration grating (TGF11, Mikromasch, Sofia, Bulgaria) was used for this purpose, and all images were obtained in ethanol. Tip radii were determined by imaging a commercially available silicon calibration grating (TGG01, Mikromasch) at 0 and 90° scan angles. The geometric mean radius of the tip was determined by the Zenhausern model of deconvolution.

After all AFM experiments, the tip radius was determined in order to normalize the results properly. This was achieved by first imaging a well-defined grating TGG01 (Mikromasch, Sofia, Bulgaria) at 0 and 90° scan angles. This grating has well-defined triangular steps with a pitch of 3 μm and apex radii of less than 10 nm, much lower than the expected radius of curvature of the AFM tips used for friction measurements. By deconvoluting the images using SPIP software by Image Metrology, it was possible to determine the tip radius from the images. The deconvolution algorithm used by SPIP is based on the blind reconstruction method described by Villarubia^62^ and Williams.^63^ Images of the TGG01 grating were obtained at a scan size of 10 × 1.25 μm with 512 samples being recorded per slow-scan axis line at a scan rate of 0.5 Hz. Three images were collected at different locations on the sample at an applied load of ca. 5 nN at the first scan angle. Once complete, the grating was rotated 90° and three more images were acquired at a scan angle perpendicular to the first. For each scan direction, the arithmetic mean radius was calculated from the three images. The final tip radius was calculated as the geometric mean of the average radius in each scan direction.

Force curves were obtained at 300 locations on each sample, repeated across three different samples and probes in each liquid. The raw NanoScope force curve data files were then imported into TToolbox for analysis.^50^ This allowed for easy batch processing of force curve files with all required tip parameters for calculation of the pull-off force. The algorithm follows the same steps as the Carpick’s Toolbox force curve Matlab routine^64^ but has been heavily optimized to speed up the calculations by 100× (or more, depending on the number of CPU threads available).

TToolbox calculates the depth of the adhesive minimum in the raw force–distance plot (in which the deflection is in units of V) from the difference between the load and unload curves at the point of separation of the tip from the surface^50^ (see the Supporting Information for further details). This quantity is then multiplied by the photodetector deflection sensitivity and the normal spring constant to yield the adhesion force. Friction–load plots were obtained by decreasing the applied load from ca. 10 nN until tip–sample separation occurred in 0.7 nN decrements, recording a 1 × 0.0625 μm^2^ friction image at each load. This process was repeated a minimum of 10 times per liquid at different locations. The average trace-minus-retrace friction signal at each line was halved and then averaged across all collected lines for each applied load.

### Sources of Error

The largest sources of experimental error are uncertainties in *F*/*R* and the possibility for site-to-site variation in the chemical composition of the surface. To minimize uncertainty in *F*/*R*, the radius of curvature of every probe used was determined. To minimize the impact of site-to-site variations in surface composition (e.g., through adventitious contamination), an algorithm was written to execute systematic random sampling on multiple different, nominally identical samples using multiple different, nominally identical probes. For each *F*/*R* value in Table 1 below, at least 2700 different force curves were acquired.

The effect of the sample roughness was considered. The Supporting Information shows an AFM height image of a typical SAM. It is important to read the height image correctly: because of the exquisite sensitivity of the AFM to changes in height, the vertical deflection can appear to change greatly when in fact it does not. Thus, the Supporting Information also shows a line section in which the height scale has equal increments to the horizontal scale. It is clear that, relative to the radius of curvature of the probe, the height changes very gradually because the gold grain size is ∼2–4 times the tip radius. Moreover, in polycrystalline gold films, the grains coalesce and their radii of curvature are significantly larger than the grain size. The only region in which the topography is likely to affect measurements significantly is at grain boundaries, where the tip experiences an increased area of contact. It is not possible to eliminate this contribution, but it will be a low-frequency occurrence, for a typical contact area with a diameter of ∼1–2 nm, and it will affect all measurements in the same fashion. Thus, we do not believe that the sample topography will systematically influence the AFM measurement data.

## Results and Discussion

### Interaction Energies in Pure Liquids

The work of adhesion *W*(Lifshitz) between two hydrocarbon surfaces in a liquid medium was calculated for 260 different liquids using Lifshitz theory (see the Supporting Information for a full list of the liquids modeled). SAMs of DDT were selected as model hydrocarbon surfaces for these calculations. Values of *W*(Lifshitz) span more than 4 orders of magnitude, from 6 × 10^–4^ mJ m^–2^ in tetramethyl silane to 25.7 mJ m^–2^ in dibutyl sulfoxide.

The interaction free energy ΔΔ*G* was calculated for the same systems using the SSIP model. In this case, the calculated values span only 2 orders of magnitude, from 0.04 kJ mol^–1^ in tetramethyl silane to 4.2 kJ mol^–1^ in sulfur dioxide.

**Figure 1:**
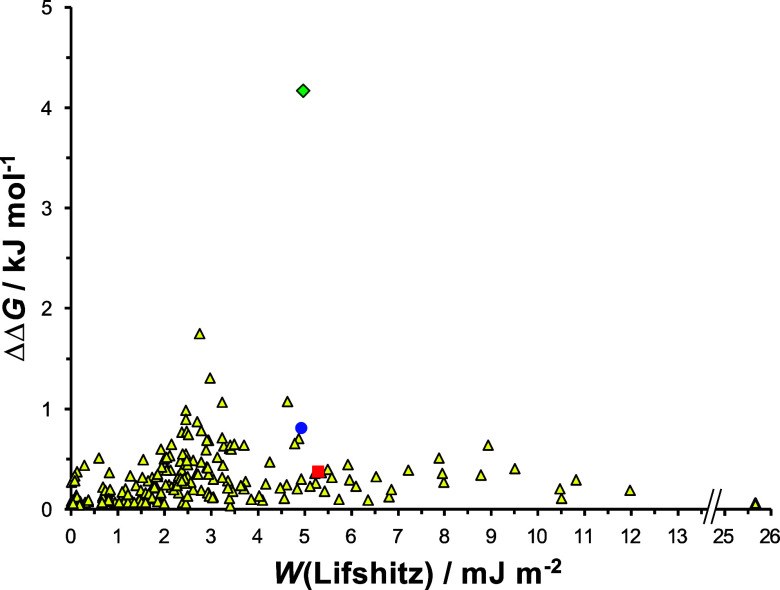
Interaction free energies ΔΔ*G* calculated using the SSIP model as a function of the work of adhesion *W*(Lifshitz) calculated using Lifshitz theory for 260 different liquids. Values are highlighted for benzyl alcohol (red square), methanol (blue circle), and water (green diamond).

**Figure 2:**
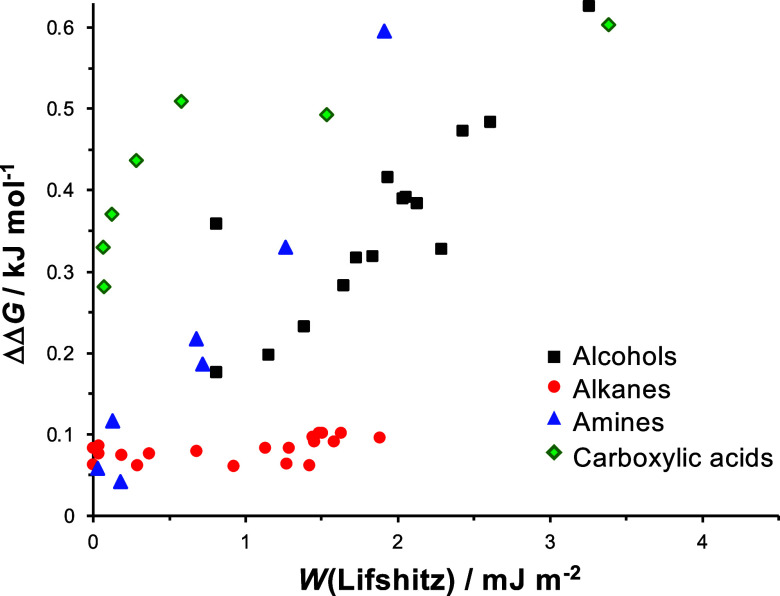
Relationship between the interaction free energy ΔΔ*G* calculated using the SSIP model and the Lifshitz work of adhesion *W*(Lifshitz) for hydrocarbon surfaces interacting in series of alkanes and polar liquids.

We conclude from the data in Figure 1 that Lifshitz theory and the SSIP model yield divergent predictions for many liquids. To examine further the predictions of these two models, data are plotted in Figure 2 for series of alkanes and for aliphatic polar liquids organized according to functional group.

The value of ΔΔ*G* is plotted as a function of *W*(Lifshitz) in Figure 1. It is striking that a large group of liquids with very different *W*(Lifshitz) values yield ΔΔ*G* values ≤0.5 kJ mol^–1^. Moreover, there are also liquids that yield similar works of adhesion but very different ΔΔ*G* values. For example, the values of *W*(Lifshitz) in benzyl alcohol, methanol, and water are 5.31 (red square in Figure 1), 4.95 (blue circle), and 4.98 (green diamond) mJ m^–2^, respectively, but the interaction free energies in the three liquids are calculated to be 0.36, 0.80, and 4.17 kJ mol^–1^, respectively. Thus, for three liquids with very similar *W*(Lifshitz) values, the smallest and largest interaction free energies differ by an order of magnitude.

The most marked difference between the predictions of the two models is observed for the alkanes (red circles in Figure 2), for which the SSIP model yields values of ΔΔ*G* that are almost invariant with *W*(Lifshitz). Large deviations are also observed between the predictions of the two models for carboxylic acids (green diamonds in Figure 2). For a number of carboxylic acids with *W*(Lifshitz) < 0.5 mJ m^–2^, ΔΔ*G* increases significantly for comparatively small changes in *W*(Lifshitz), but a limiting value of ΔΔ*G* is reached for *W*(Lifshitz) > 0.5 mJ m^–2^.

More subtle differences are observed for alcohols and amines. For both these series of liquids, the interaction free energy is proportional to the work of adhesion. However, the constant of proportionality is clearly different for these two series of liquids, indicating that there is divergence between the predictions of the two models. The work of adhesion can be estimated from the ΔΔ*G* data by using the relationship *W*(SSIP) ∼ ΔΔ*G*/σ, where σ is the area occupied by an adsorbate in a DDT SAM.^65^ Thus, we estimate that *W*(SSIP) ∼ 5/2 *W*(Lifshitz) for amines, representing a substantial difference between the predictions of the two models.

To test the interaction energies calculated using the Lifshitz and SSIP models against experimental data, measurements of pull-off forces were made by AFM for DDT SAMs interacting in a representative selection of pure liquids that included both aliphatic and aromatic solvents and polar and nonpolar liquids. A silicon nitride probe was coated with a thin layer of gold, and a SAM of DDT was formed by immersion of the probe in a dilute solution of the thiol in ethanol. A counter surface was prepared by forming a DDT SAM on a continuous polycrystalline gold film supported on a glass substrate, and the tip–sample adhesion force *F*_po_ was measured. Values of *F*_po_ are shown in Table 1 for an illustrative selection of liquids with varying dielectric constants. It is important to note that measurements by AFM are constrained by both the physical properties of the liquids (liquids with very small surface tensions are difficult to handle in the AFM liquid cell) and also the associated hazards (evaporation occurs from the liquid cell and the risk of exposure to vapor needs to be considered carefully).

To enable comparison of these experimental data with predictions made using the Lifshitz and SSIP models, the experimental work of adhesion was calculated from the *F*_po_ values. If the tip–sample contact is described using the Derjaguin–Muller–Toporov model of contact mechanics, the pull-off force is related to the experimental work of adhesion *W*(exp) by^1^
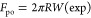
14

The resulting experimental works of adhesion are displayed in Table 1 together with calculated values determined using the Lifshitz and SSIP models. The Lifshitz works of adhesion are those plotted in Figure 1 and tabulated in the Supporting Information. The SSIP work of adhesion was estimated using the relationship *W*(SSIP) ∼ ΔΔ*G*/σ.

The most striking difference between the two models is observed in water. The Lifshitz work of adhesion for DDT SAMs in this liquid, 4.98 mJ m^–2^, is nearly an order of magnitude smaller than the experimental value, 46.5 ± 1.9 mJ m^–2^. In contrast, the value of *W*(SSIP), 37.08 mJ m^–2^, is close to the experimental work of adhesion. There are two reasons for this large discrepancy between the predictions of the two models. First, water is not only a strongly polar liquid, but it possesses strong, directional noncovalent bonds (hydrogen bonds). Second, because the SSIP treatment is based on a thermodynamic treatment of works of adhesion, it is able to account for solvophobic effects (in this specific case, hydrophobicity) in a way that the Lifshitz model cannot.

For methanol, the Lifshitz work of adhesion, 4.95 mJ m^–2^, is similar to the value calculated in water, whereas *W*(SSIP), 7.19 mJ m^–2^, is much closer to the experimental value of 6.52 ± 0.53 mJ m^–2^. For ethanol, both models correctly yield a work of adhesion that is smaller than that in methanol, but again, the *W*(SSIP) value, 5.61 mJ m^–2^, is much closer to the experimentally determined value, 5.41 ± 0.63 mJ m^–2^, than *W*(Lifshitz), 3.26 mJ m^–2^.

Both models overestimate the work of adhesion in the aromatic liquids, benzyl alcohol and benzonitrile. However, the SSIP model yields values that are closest to the experimental data in both liquids and correctly predicts that the work of adhesion will be substantially larger in benzyl alcohol than in benzonitrile, whereas the Lifshitz model incorrectly yields very similar works of adhesion for these two liquids.

For the remaining five liquids, the Lifshitz model yields values that span 2 orders of magnitude, from 0.00 mJ m^–2^ for *n*-dodecane and 0.04 mJ m^–2^ for *n*-decane and *n*-hexadecane, to 0.39 mJ m^–2^ for *n*-heptane and 6.36 mJ m^–2^ for 1,2,4-trichloro-benzene. In sharp contrast, the SSIP model yields values that range from 0.72 mJ m^–2^ for *n*-heptane to 0.81 mJ m^–2^ for 1,2,4-trichloro-benzene. The experimental works of adhesion span a slightly larger range, from 0.27 ± 0.16 to 0.60 ± 0.18 mJ m^–2^, but bearing in mind the experimental uncertainty, the behavior is broadly consistent with the predictions of the SSIP model; certainly, these data do not display the orders-of-magnitude changes predicted by the Lifshitz model.

**Figure 3:**
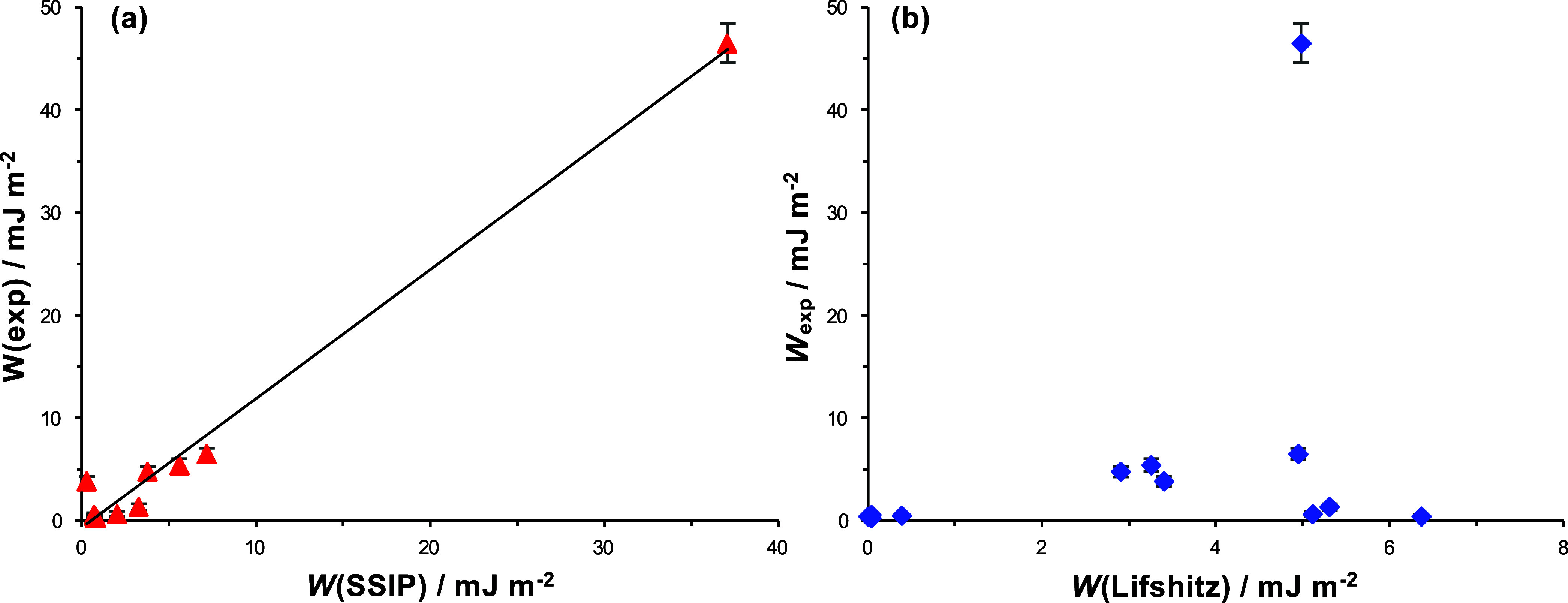
Experimental work of adhesion data from Table 1 displayed as a function of (a) work of adhesion calculated using the SSIP model and (b) Lifshitz work of adhesion.

Figure 3 shows the experimental work of adhesion data from Table 1*W*(exp) as a function of *W*(SSIP) and *W*(Lifshitz). While the value of *W*_exp_ increases with *W*(SSIP) (a regression coefficient of 0.84 is obtained for the straight line fit in Figure 3a), there is no correlation between the value of *W*(Lifshitz) and *W*(exp) in Figure 3b.

In summary, works of adhesion determined experimentally from AFM adhesion force measurements are correlated closely with interaction free energies calculated using the SSIP model. In contrast, works of adhesion calculated using the Lifshitz model are not well correlated with the experimental data.

### Methanol and Benzyl Alcohol: Nanotribological Measurements

**Figure 4:**
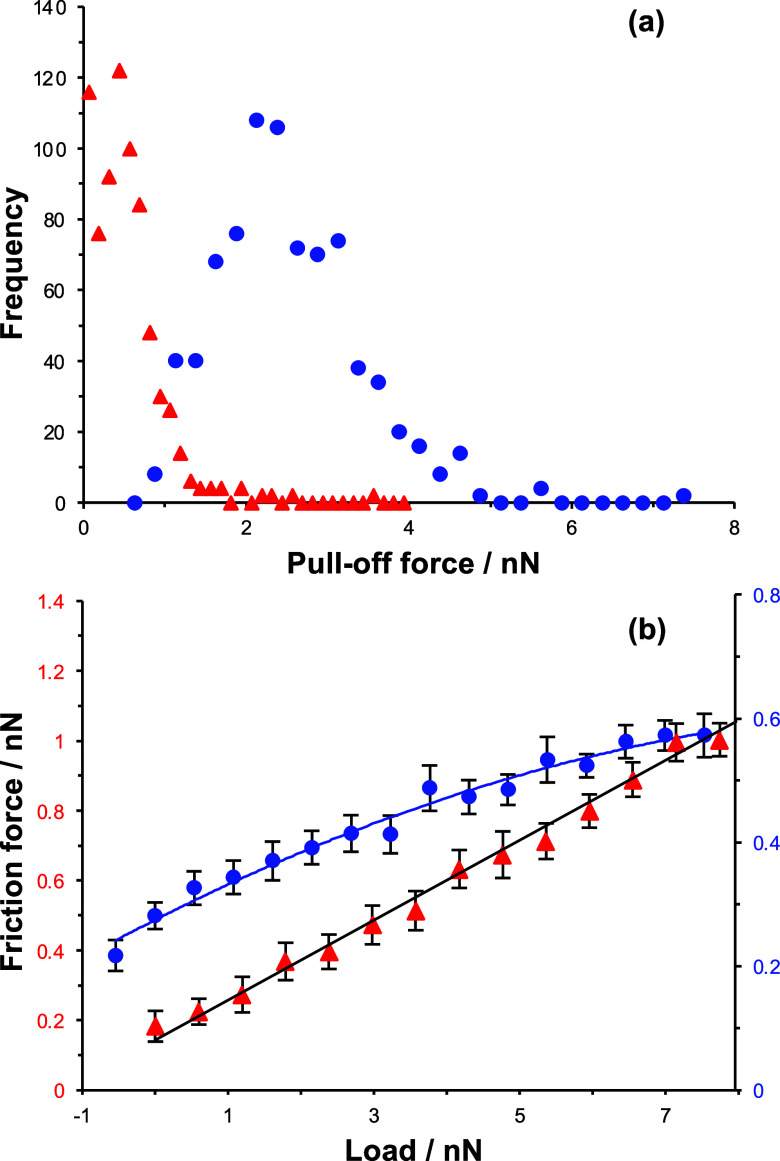
Pull-off force frequency distributions (a) and friction–load relationships (b) for DDT SAMs interacting in benzyl alcohol (red triangles) and methanol (blue circles). Lines in (b) are fitted using eq 15.

Using the Lifshitz model, we calculated that the work of adhesion for interacting DDT SAMs is slightly larger (5.31 mJ m^–2^) in pure benzyl alcohol than in pure methanol (4.95 mJ m^–2^). In contrast, using the SSIP model, we calculated interaction energies of 3.28 and 7.19 mJ m^–2^, respectively. Thus, the two models yield significantly different predictions for these pure liquids. Histograms of pull-off forces *F*_po_ are shown in Figure 4a. In benzyl alcohol (red triangles), the pull-off force peaks at small values, and the distribution of forces is narrow. However, in methanol, the distribution of forces is broader and the maximum in the frequency distribution lies between 2.1 and 2.4 nN, indicating a significantly stronger adhesion force in this liquid, consistent with the predictions of the SSIP model and contrary to the predictions of the Lifshitz model.

Friction–load relationships were acquired for DDT-functionalized AFM probes in contact with DDT SAMs. Following the work of Bowden and Tabor, Carpick, and others, we treat the friction force *F*_F_ in the sliding contact as the sum of two terms, an area-dependent shear term characterized by a surface shear strength τ and a load-dependent term attributed to “molecular plowing” characterized by a coefficient of friction μ^42,44,56^
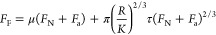
15where *F*_N_ is the load perpendicular to the planar counter surface, *F*_a_ is the adhesion force, *R* is the radius of the probe, and *K* is the elastic modulus of the materials in contact. In studies of a variety of materials, including SAMs and surface-grafted polymers, we showed that for solvated interfaces, the work of adhesion is typically small, and sliding is dominated by molecular plowing; thus, the shear term becomes small and the friction–load relationship is linear.^39−41,56^ In this regime, energy dissipation is largely through the deformation of molecules under the probe (e.g., through the creation of gauche defects in SAMs). However, as the interface becomes increasingly less well solvated, the shear term begins to make an important contribution to friction; energy is increasingly dissipated in shearing and the friction–load relationship becomes nonlinear. Thus, in general, a linear friction–load relationship indicates weak adhesion (dissipation dominated by plowing) and a nonlinear friction–load relationship indicates strong adhesion (dissipation dominated by shearing).

Figure 4b shows friction–load relationships acquired for DDT contacts in methanol and benzyl alcohol. It is clear that while the friction–load relationship is linear in benzyl alcohol, with a coefficient of friction μ = 0.11 ± 0.02, it is nonlinear in methanol. While the shear term is negligible after fitting friction–load data measured in benzyl alcohol, it is the load-dependent term that is negligible in methanol, and fitting of the curve yields a surface shear strength τ/*K*^2/3^ = 2.17 ± 0.43 Pa^2/3^. This indicates that in benzyl alcohol, the main dissipative pathway is via plowing (correlated with weak adhesion), whereas in methanol, shearing dominates, consistent with strong adhesion. This qualitative difference in the nature of the friction–load relationship is consistent with the predictions of the SSIP model and refutes the prediction based on the Lifshitz model that adhesion is similar for contacts in methanol and benzyl alcohol.

### Interaction Energies in Mixtures of Methanol and Benzyl Alcohol

**Figure 5:**
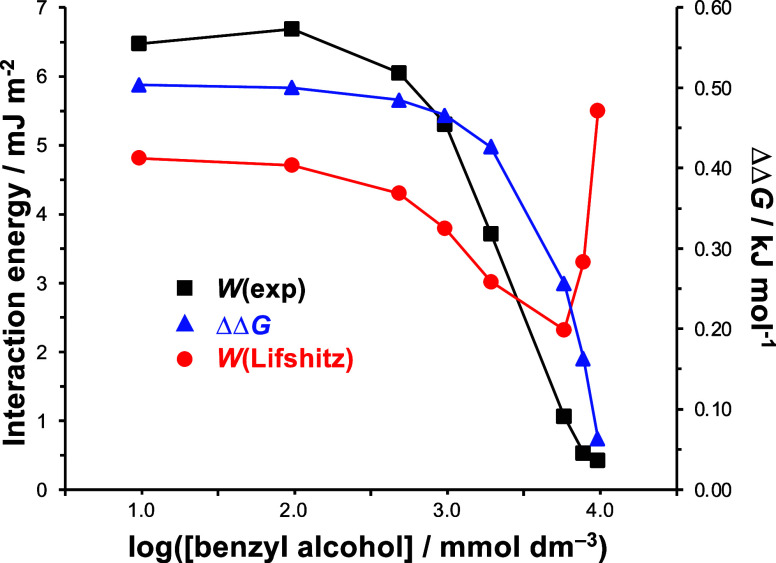
Interaction energies determined experimentally from AFM force measurements (black squares) and by calculation using the Lifshitz (red circles) and SSIP (blue triangles) models for mixtures of benzyl alcohol and methanol, with mole fractions of benzyl alcohol ranging from 0.01 to 1.0.

To test further the predictive capabilities of the Lifshitz and SSIP models, we calculated interaction energies for hydrocarbon surfaces in mixtures of methanol and benzyl alcohol. Data are shown in Figure 5. The concentration of benzyl alcohol is shown on the horizontal axis. In the SSIP model (blue triangles), as log([benzyl alcohol]/mmol dm^–3^) increases from 1 to 2.5, the interaction free energy changes comparatively little. However, as log([benzyl alcohol]/mmol dm^–3^) is increased above 2.5, the interaction free energy begins to decrease and falls steeply as log([benzyl alcohol]/ mmol dm^−3^) decreases from 3.8 to 4.0. This behavior can be understood in terms of the solvent–surface interaction: methanol interacts weakly with the hydrocarbon surfaces and only perturbs the adhesive interaction in a small way; in contrast, benzyl alcohol interacts more strongly with the DDT SAMs, coordinating to them more extensively and shifting the equilibrium in the direction of a solvated interface, thus reducing the strength of adhesion between the probe and counter surface when they interact.

Works of adhesion were also calculated using the Lifshitz model (red circles). Between log([benzyl alcohol]/mmol dm^–3^) of 1 and 2.5, the work of adhesion decreases slowly, but at higher concentrations of benzyl alcohol, the work of adhesion begins to decrease more rapidly, mirroring the behavior of the SSIP model. However, in contrast to the SSIP model, a minimum is reached in the Lifshitz work of adhesion between log([benzyl alcohol]/mmol dm^–3^) = 3.3 and 3.8, and at higher benzyl alcohol concentrations, the work of adhesion increases sharply, reaching a value at log([benzyl alcohol]/mmol dm^–3^) = 4.0 that is larger than the value obtained at the lowest benzyl alcohol concentration.

Figure 5 shows experimental work of adhesion data acquired by AFM using the previously described methodology (black squares). These data match the trend predicted by the SSIP model, although the experimental work of adhesion *W*(exp) decreases in magnitude slightly more quickly as the concentration of benzyl alcohol is increased. The value of *W*_exp_ reaches a minimum in pure benzyl alcohol, as predicted by the SSIP model and in contrast to the Lifshitz model, which predicts a maximum value in this liquid. Thus, we conclude that for mixtures of benzyl alcohol and methanol, the experimental data are predicted significantly more reliably by the SSIP model than by the Lifshitz model.

These data further support the hypothesis that works of adhesion determined experimentally from AFM adhesion force measurements are correlated more closely with interaction free energies calculated using the SSIP model than with works of adhesion calculated using the Lifshitz model.

## Conclusions

We calculated interaction energies for hydrocarbon surfaces in 260 liquids using Isrealchvilli’s modified form of the Lifshitz theory and Hunter’s SSIP model. Values of the work of adhesion calculated using Lifshitz theory spanned a wide range, from 6 × 10^–4^ mJ m^–2^ in tetramethyl silane to 25.7 mJ m^–2^ in dibutyl sulfoxide. However, the SSIP model predicts much smaller differences in the interaction free energy for the majority of these liquids. When these predictions are compared with measurements made using AFM, the SSIP approach is found to yield works of adhesion that are significantly closer to the experimental data than the predictions made using the Lifshitz model. For some liquids for which Lifshitz theory predicts similar works of adhesion, the SSIP model predicts very different values. In these cases, the experimental data are consistent with the predictions of the SSIP model and are not correlated with the predictions made using the Lifshitz model. For methanol, dissipation in sliding contacts between hydrocarbon monolayers is dominated by shearing, while in benzyl alcohol, dissipation is through molecular plowing. These differences are consistent with the large difference in work of adhesion predicted using the SSIP model, and with measurements of adhesion forces, while in contrast, the Lifshitz model predicts that the works of adhesion measured in these two liquids are very similar. In methanol/benzyl alcohol mixtures, works of adhesion calculated using the Lifshitz model pass through a minimum and approach a maximum in pure benzyl alcohol, whereas experimental pull-off force values and works of adhesion calculated using the SSIP model decline to reach a minimum in pure benzyl alcohol. We conclude that the use of mean bulk dielectric properties to calculate interaction energies using the Lifshitz model represents a significant and under-appreciated limitation. A molecular approach based upon a thermodynamic analysis of interfacial equilibria using the SSIP model yields predictions that are more reliable and much closer to experimental data.

## Supporting Information Available

XPS and AFM data for SAMs, dielectric constants, refractive indices, and Lifshitz works of adhesion and interaction free energies for DDT SAMs in 260 liquids (PDF)
