# Supplementary material for: Transcending Lifshitz Theory: Reliable Prediction of Adhesion Forces between Hydrocarbon Surfaces in Condensed Phases Using Molecular Contact Thermodynamics
Source: Langmuir. 2024 Jun 27;40(27):13753–62. doi: 10.1021/acs.langmuir.3c03218 (PMC11238592; doi:10.1021/acs.langmuir.3c03218)
Supplement: Supplementary file 1 — la3c03218_si_001.pdf [file la3c03218_si_001.pdf]

Supplementary Information

Transcending Lifshitz Theory: Reliable Prediction of Adhesion Forces between Hydrocarbon Surfaces in Condensed Phases using Molecular Contact Thermodynamics

Oscar Siles Brügge,[1†] Christopher A Hunter[2] and Graham J Leggett[1]

[1]*Department of Chemistry, University of Sheffield, Brook Hill, Sheffield S3 7HF, UK and*
[2]*Department of Chemistry, University of Cambridge, Lensfield Road, Cambridge CB2 1EW, UK;*
†Present address: Department of Chemistry, University of Nottingham, University Park, Nottingham NG7 2RD.

*Corresponding Author. E-mail: Graham.Leggett@sheffield.ac.uk

Characterisation of Self-Assembled Monolayers

Self-assembled monolayers (SAMs) of dodecanethiol (DDT) on gold surfaces were characterised by X-ray photoelectron spectroscopy (XPS). The C1s spectrum (Figure S1) displayed a single peak at 285.0 eV corresponding to the carbon atoms in the alkyl chain of the adsorbate, and the S2p spectrum displayed a doublet (due to spin-orbit coupling) corresponding to the alkylthiolate sulfur atom, consistent with previously published literature.[1, 2] The absence of a sulfonate peak around 168 – 170 eV demonstrates that the SAMs used for analysis have not been oxidized after deposition and storage.[2, 3]

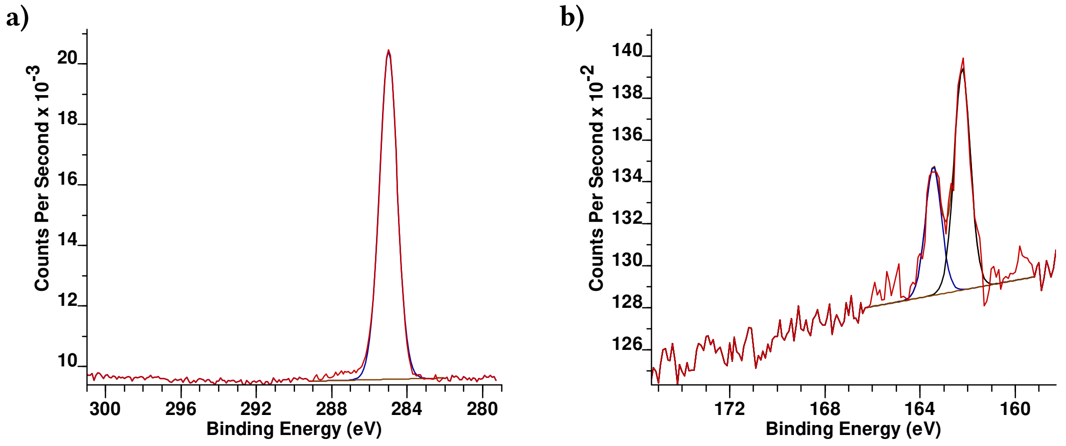

Figure S1. High resolution XPS C1s (a) and S2p (b) spectra of a self-assembled monolayer formed by the adsorption of dodecanthiol on gold.

Every sample used for force measurements was first characterised by measuring the advancing water contact angle. The mean value of the contact angle was found to be 112 ± 2º, consistent with published values.[1] Samples for which the contact angle deviated significantly from this value were discarded.

The surface roughness of SAMs on planar surfaces was determined using tapping mode AFM. A tip of radius 34 ± 3 nm was used. One of the topographical images recorded for these systems is displayed in Figure S2, with the average of several of these images (across various samples) giving an average roughness (R_a) value of 2.95nm and a root mean square deviation (R_q) of 3.73 nm. The ratio of the two (R_q / R_a) resulted in a factor of 1.264, indicating a Gaussian asperity height distribution, due to its proximity to the ideal value of 1.253 for such a distribution. The gold grain size was in the range 20 - 80 nm for these samples.

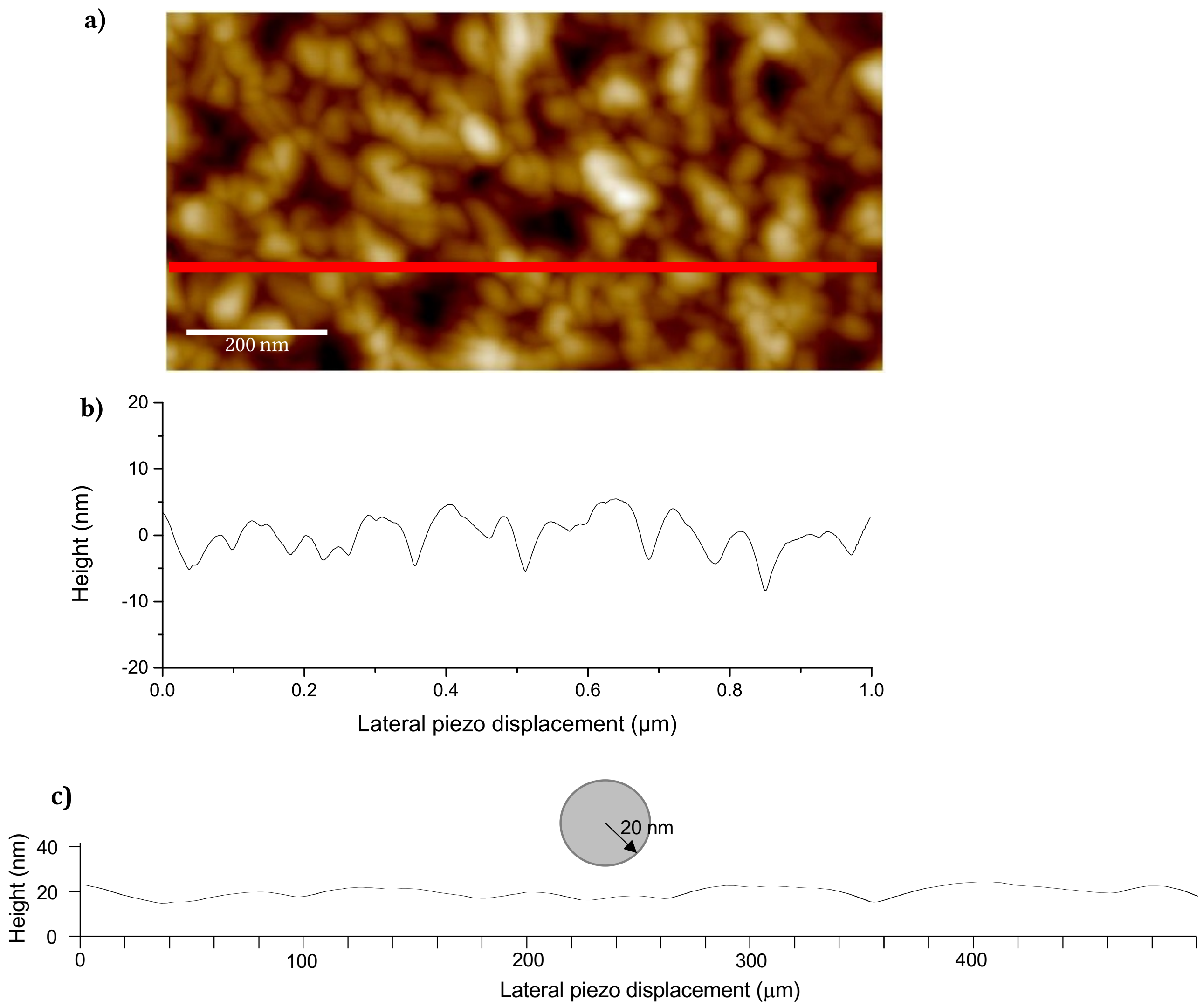

Figure S2. (a) Typical height image of a DDT SAM acquired using tapping mode AFM. (b) Section along the red line in (a). (c) Section with equal increments on horizontal and vertical scales, together with a circle of equal radius to a typical probe, to illustrate the roughness relative to the probe dimensions.

Adhesion Force Measurements

Force curves were obtained for at least 300 locations on each sample, repeated across three different samples and probes in each liquid. Figure S3a shows a typical force curve. Using the algorithm in TToolbox,[4] it was possible to acquire thousands of force curves in each liquid in an automated fashion.

To determine pull-off forces, the magnitude of the difference in the photodetector signal between the approach and retract curves was determined in the region of negative (adhesive) signal. The approach curve, as the probe is brought into contact with the surface, was not modelled; thus, pull-off forces reported herein are equal in magnitude to the depth of the minimum in the adhesive portion of the pull-off curve. Using the force curve in Figure S3 as an example, the depth of the force minimum was found to be –0.0865 V. This is then multiplied by the photodetector deflection sensitivity and the normal spring constant to yield the force F_{po} required to separate the tip from the surface:

$$F_{po} = 0.0865\ \text{V} \times 0.1230\ \text{Nm}^{-1} \times 48.75\text{nm V}^{-1} = 0.5187\text{nN}$$

This calculation was performed for at least 300 force curves recorded at different locations, and repeated for at least three different samples, after which the mean value of F_{po} was determined.

Using these data, histograms of adhesion forces (i.e. F_{po}) were plotted for each liquid studied. Typical examples of such histograms, acquired for contacts between DDT-functionalised probes and DDT-functionalised counter-surfaces in water and heptane are shown in Figures S3b and S3c, respectively.

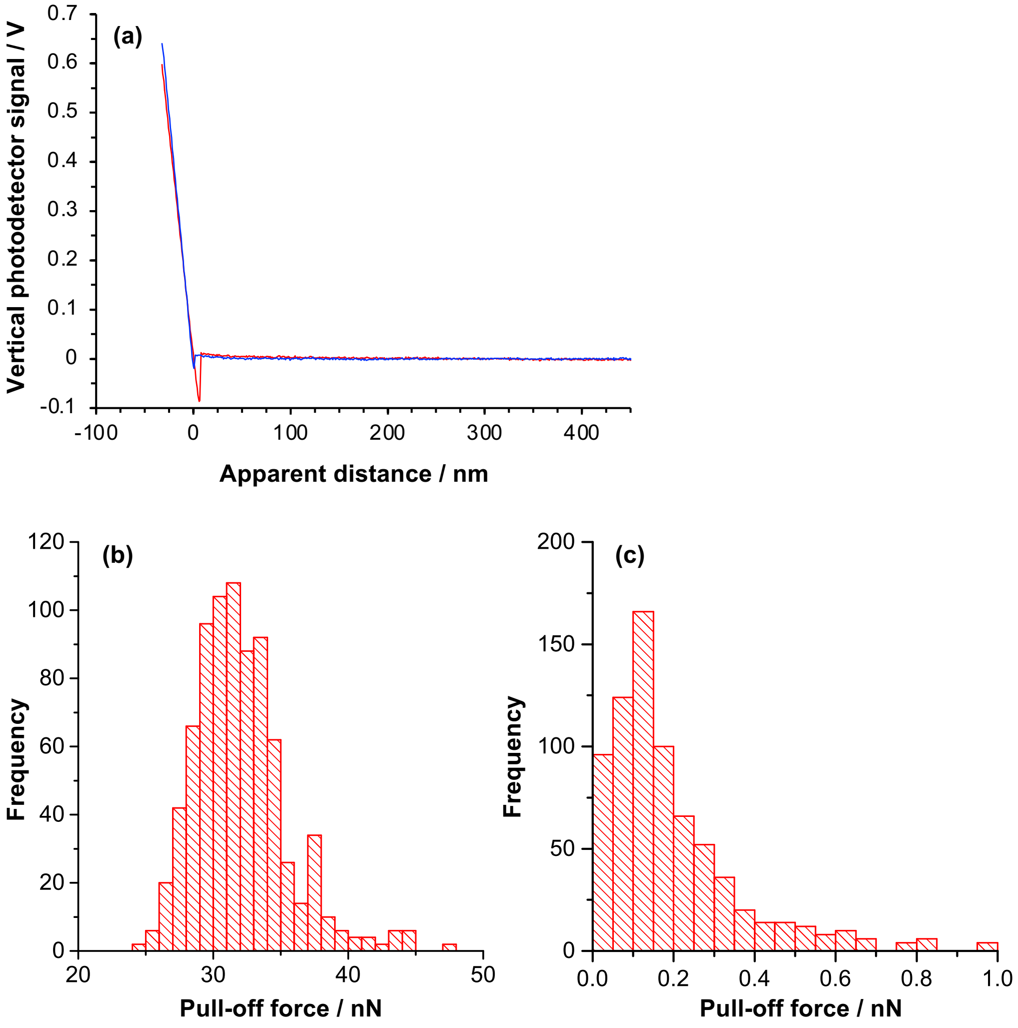

Figure S3. (a) Force curve obtained with 1-dodecanethiol SAM functionalized AFM probe and sample in ethanol. The blue line shows the approach signal, while the red shows the retract signal used for determination of the pull-off force. (b,c) Examples of histograms showing the distribution of adhesion forces measured for DDT SAMs in (b) water and (c) heptane.

Table S1 Calculated values of W(Lifshitz) and $\Delta\Delta G$ (SSIP) for 260 liquids. ε, n_D average values at 20°C obtained from Marcus et al.[5] and Lide et al.[6]

Medium	ε	n	W_{3med} (mJ m^{-2})	W_{5med} (mJ m^{-2})	$\Delta G°$ (kJ mol^{-1})
tetramethylsilane	1.92	1.358	1.135	1.145	0.083
n-pentane	1.84	1.355	1.270	1.270	0.064
2-methylbutane	1.83	1.351	1.422	1.420	0.062
n-hexane	1.88	1.372	0.681	0.686	0.079
cyclohexane	2.02	1.424	0.002	0.000	0.063
n-heptane	1.92	1.385	0.368	0.378	0.076
n-octane	1.95	1.395	0.191	0.204	0.074
2_2_4-trimethylpentane	1.96	1.389	0.292	0.306	0.062
n-decane	1.99	1.410	0.036	0.055	0.076
n-dodecane	2	1.420	0.001	0.000	0.083
n-hexadecane	2.05	1.433	0.038	0.063	0.086
benzene	2.27	1.498	1.630	1.680	0.102
toluene	2.38	1.494	1.487	1.547	0.102
ortho-xylene	2.57	1.503	1.883	1.961	0.095
meta-xylene	2.37	1.495	1.505	1.565	0.102
para-xylene	2.27	1.493	1.445	1.495	0.097
ethylbenzene	2.40	1.493	1.453	1.516	0.091
isopropylbenzene	2.38	1.489	1.288	1.349	0.083
1_3_5-trimethylbenzene	2.28	1.497	1.585	1.636	0.091
styrene	2.43	1.544	4.097	4.162	0.090
1-chloro- 1_2_3_4-tetrahydronaphthalene	2.77	1.539	3.842	3.936	0.093
cis-decalin	2.20	1.479	0.927	0.970	0.061
water	78.36	1.333	4.984	5.026	4.167
methanol	32.66	1.327	4.945	5.034	0.804
ethanol	24.55	1.359	3.258	3.367	0.626
1-propanol	20.45	1.384	2.426	2.550	0.473
2-propanol	19.92	1.375	2.608	2.734	0.483
1-butanol	17.51	1.397	2.057	2.193	0.391
2-methyl-1-propanol	17.93	1.394	2.128	2.263	0.384
2-butanol	16.56	1.395	2.037	2.178	0.390
2-methyl-2-propanol	12.47	1.385	1.938	2.101	0.416
1-pentanol	13.90	1.408	1.731	1.886	0.317
3-methyl-1-butanol	15.19	1.405	1.840	1.988	0.318
2-methyl-2-butanol	5.78	1.402	0.812	0.996	0.358
1-hexanol	13.30	1.416	1.647	1.805	0.283
cyclohexanol	15	1.465	2.289	2.437	0.327
1-octanol	10.34	1.428	1.385	1.560	0.232
1-decanol	8.10	1.435	1.151	1.337	0.197
1-dodecanol	5.70	1.441	0.809	0.993	0.176
benzyl_alcohol	12.70	1.538	5.306	5.468	0.363
2-phenylethanol	12.31	1.533	4.918	5.081	0.299
allyl_alcohol	21.60	1.411	2.099	2.219	0.532
2-chloroethanol	25.80	1.442	2.327	2.432	0.476
2-cyanoethanol					0.566
2_2_2-trifluoroethanol	26.67	1.291	7.213	7.316	0.384
1_1_1_3_3_3-hexafluoro- 2-propanol	16.62	1.277	7.970	8.111	0.263
2-methoxyethanol	16.93	1.400	1.992	2.131	0.499
2-ethoxyethanol	29.60	1.406	2.359	2.454	0.385
ethylene_glycol	37.70	1.431	2.456	2.535	0.984

Continued on next page

Table S1 *continued*

Medium	ε	n	$W_{3\text{med}}$ (mJ m^{-2})	$W_{5\text{med}}$ (mJ m^{-2})	$\Delta G°$ (kJ mol^{-1})
1_2-propanediol	32	1.431	2.370	2.460	0.761
1_3-propanediol	35	1.439	2.477	2.561	0.775
1_2-butanediol		1.437			0.605
2R_3S-butanediol	21.53	1.437	2.144	2.263	0.645
1_4-butanediol	30.20	1.444	2.456	2.550	0.897
1_5-pentanediol		1.448			0.745
diethylene_glycol	31.69	1.446	2.510	2.600	0.739
triethylene_glycol	23.69	1.454	2.447	2.559	0.544
glycerol	42.50	1.473	3.236	3.307	0.708
phenol	11.60	1.543	5.482	5.650	0.396
ortho-cresol	11.50	1.544	5.570	5.738	0.314
meta-cresol	12.44	1.540	5.360	5.523	0.360
para-cresol	11.07	1.539	5.205	5.376	0.352
2_6-dimethoxyphenol					0.341
6-amino-2_4-dimethylphenol	6.16	1.525	3.733	3.919	0.271
3-chlorophenol		1.563			0.350
diethyl_ether	4.20	1.350	1.852	2.013	0.096
di-n-propyl_ether	3.39	1.378	0.728	0.859	0.072
diisopropyl_ether	3.88	1.366	1.190	1.342	0.079
dibutyl_ether	3.08	1.397	0.302	0.417	0.068
bis2-chloroethyl_ether	21.20	1.455	2.385	2.506	0.068
1_2-dimethyoxyethane	7.20	1.378	1.486	1.673	0.187
diethylene_glycol_dimethyl_ether	5.80	1.406	0.779	0.963	0.149
furan	2.94	1.419	0.110	0.216	0.134
tetrahydrofuran	7.58	1.405	1.092	1.279	0.173
2-methyltetrahydrofuran	5.26	1.405	0.677	0.858	0.130
tetrahydropyran	5.61	1.419	0.678	0.861	0.116
1_3-dioxane	2.21	1.420	0.008	0.051	0.267
1_3-dioxolan		1.399			0.322
1_8-cineole	4.57	1.456	0.791	0.960	0.075
anisole	4.33	1.514	2.774	2.939	0.189
ethyl_phenyl_ether	4.22	1.505	2.304	2.465	0.164
diphenyl_ether	3.60	1.578	6.797	6.938	0.117
dibenzyl_ether	3.86	1.539	4.023	4.174	0.132
1_2-dimethoxybenzene	4.09	1.532	3.700	3.858	0.200
methyl_orthoformate		1.379			0.174
methyl_orthoacetate		1.381			0.147
propionaldehyde	18.50	1.359	3.041	3.172	0.295
butyraldehyde	13.40	1.377	2.210	2.367	0.235
benzaldehyde	17.80	1.544	5.963	6.098	0.287
p-methoxybenzaldehyde	15.50	1.573	7.933	8.079	0.354
cinnamaldehyde	16.90				0.303
acetone	20.56	1.356	3.249	3.372	0.438
2-butanone	18.11	1.377	2.484	2.618	0.318
2-pentanone	15.38	1.389	2.082	2.228	0.242
3-methyl-2-butanone	15.87				0.241
3-pentanone	17	1.390	2.146	2.285	0.217
cyclopentanone	14.45	1.435	1.779	1.931	0.257
4-methyl-2-pentanone	13.11	1.394	1.838	1.997	0.207
3_3-dimethyl-2-butanone	12.60	1.395	1.776	1.938	0.216
perfluorooctane					−0.005
cyclohexanone	15.50	1.450	2.023	2.169	0.239

Continued on next page

Table S1 *continued*

**Medium**	ε	n	$W_{3\text{med}}$ (mJ m^{-2})	$W_{5\text{med}}$ (mJ m^{-2})	ΔG° (kJ mol^{-1})
2-heptanone	11.98	1.407	1.590	1.756	0.165
3-heptanone	12.88	1.407	1.665	1.826	0.154
2_2_4_4-tetramethyl-3-pentanone	14.50	1.420	1.725	1.876	0.111
acetophenone	17.39	1.532	5.227	5.364	0.257
ethyl_phenyl_ketone	15.50	1.527	4.829	4.975	0.204
benzyl_methyl_ketone		1.517			0.228
2_4_5-trimethylacetophenone		1.534			0.225
p-chloroacetophenone	9.60	1.555	6.099	6.277	0.223
diphenyl_ketone	11.40	1.606	10.467	10.636	0.202
2_4-pentanedione	25.70	1.447	2.381	2.487	0.546
2_3-butanedione		1.393			0.541
formic_acid	58.50	1.369	3.383	3.438	0.603
acetic_acid	6.15	1.370	1.530	1.716	0.493
propanoic_acid	3.37	1.384	0.579	0.710	0.510
butanoic_acid	2.90	1.396	0.282	0.385	0.437
pentanoic_acid	2.66	1.406	0.124	0.209	0.371
hexanoic_acid	2.63	1.415	0.067	0.149	0.330
heptanoic_acid	2.71	1.421	0.068	0.158	0.282
cis-perfluorodecalin	1.98	1.313	3.403	3.420	0.029
fluorobenzene	5.42	1.462	1.104	1.286	0.076
hexafluorobenzene	2.05	1.374	0.632	0.658	0.044
1_4-dichlorobutane	7.39	1.400	1.115	1.302	0.063
chlorobenzene	5.62	1.521	3.391	3.574	0.101
dichloromethane	8.93	1.421	1.208	1.390	0.062
1_1-dichloroethane	10	1.413	1.354	1.530	0.061
1_2-dichloroethane	10.36	1.442	1.498	1.673	0.066
trans-1_2-dichloroethylene	2.14	1.446	0.178	0.214	0.077
ortho-dichlorobenzene	9.93	1.549	5.725	5.902	0.095
meta-dichlorobenzene	5.04	1.543	4.564	4.742	0.105
chloroform	4.89	1.442	0.652	0.827	0.062
1_1_1-trichloroethane	7.25	1.435	1.023	1.210	0.064
1_1_2-trichloroethane	7.29	1.468	1.585	1.773	0.062
trichloroethylene	3.42	1.475	1.011	1.144	0.068
1_2_4-trichlorobenzene	4.15	1.571	6.356	6.516	0.086
carbon_tetrachloride	2.24	1.457	0.370	0.416	0.086
tetrachloroethylene	2.28	1.503	1.849	1.900	0.069
1_1_2_2-tetrachloroethane	8.20	1.491	2.454	2.639	0.053
pentachloroethane	3.73	1.500	1.980	2.126	0.055
iodobenzene	4.49	1.617	10.488	10.656	0.102
methylene_iodide	5.32	1.738	25.668	25.849	0.055
n-butylamine	4.88	1.398	0.677	0.852	0.218
benzylamine	4.60	1.538	4.156	4.326	0.252
ethylenediamine	12.90	1.454	1.911	2.071	0.595
diethylamine	3.78	1.382	0.719	0.867	0.187
di-n-butylamine	2.98	1.415	0.127	0.235	0.117
pyrrole	8.13	1.507	3.117	3.302	0.518
pyrrolidine		1.440			0.227
piperidine	5.80	1.452	0.981	1.165	0.197
morpholine	7.42	1.452	1.262	1.450	0.330
triethylamine	2.42	1.398	0.177	0.242	0.043
tri-n-butylamine	2.29	1.428	0.028	0.080	0.059
aniline	6.98	1.583	7.878	8.065	0.506

Continued on next page

Table S1 *continued*

Medium	ε	n	W_{3med} (mJ m^{-2})	W_{5med} (mJ m^{-2})	$\Delta G°$ (kJ mol^{-1})
o-chloroaniline	13.40	1.585	8.767	8.925	0.339
methylphenylamine	6.06	1.568	6.521	6.707	0.326
N_N-dimethylaniline	4.91	1.556	5.412	5.588	0.174
aminoethanol	37.72	1.452	2.698	2.776	0.871
diethanolamine	25.19	1.473	2.933	3.040	0.682
triethanolamine	29.36	1.483	3.345	3.441	0.285
pyridine	12.91	1.507	3.626	3.786	0.232
2-methylpyridine	9.80	1.498	2.935	3.113	0.163
3-methylpyridine	11.35	1.506	3.445	3.614	0.177
4-methylpyridine	11.86	1.503	3.357	3.523	0.208
2_4-dimethylpyridine	9.60	1.498	2.912	3.091	0.158
2_6-dimethylpyridine	7.33	1.495	2.481	2.668	0.131
2_4_6-trimethylpyridine	12.02	1.495	3.035	3.200	0.118
2-cyanopyridine	93.80	1.529	5.914	5.949	0.441
pyrimidine		1.499			0.367
quinoline	8.95	1.624	11.957	12.139	0.184
acetonitrile	35.94	1.341	4.255	4.337	0.468
propionitrile	28.26	1.363	3.228	3.327	0.313
n-butyronitrile	24.83	1.382	2.610	2.718	0.189
3-methylbutanenitrile	19.71	1.395	2.192	2.318	0.191
acrylonitrile	33	1.388	2.667	2.755	0.352
phenylacetonitrile	18.70	1.520	4.615	4.746	0.240
benzonitrile	25.20	1.525	5.116	5.223	0.223
nitromethane	35.87	1.379	2.907	2.989	0.422
nitroethane	28.06	1.389	2.550	2.649	0.265
1-nitropropane	23.24	1.399	2.265	2.379	0.232
2-nitropropane	25.52	1.392	2.434	2.540	0.258
nitrobenzene	34.78	1.550	6.852	6.936	0.192
formamide	109.50	1.446	2.968	2.998	1.300
N-methylformamide	182.40	1.430	2.899	2.918	0.686
N_N-dimethylformamide	36.71	1.428	2.430	2.510	0.451
N_N-dimethylthioformamide	47.50	1.576	8.922	8.987	0.638
N_N-diethylformamide	29.02	1.434	2.327	2.424	0.306
N-methylacetamide	191.30	1.425	2.887	2.905	0.588
N_N-dimethylacetamide	37.78	1.435	2.486	2.565	0.445
N_N-diethylacetamide	31.33	1.439	2.418	2.509	0.302
2-pyrrolidinone	27.79	1.486	3.413	3.513	0.594
N-methyl_pyrrolidinone	32.20	1.467	2.929	3.018	0.345
N-methyl_thiopyrrolidinone	47.50	1.583	9.493	9.558	0.405
tetramethylurea	23.60	1.449	2.358	2.470	0.285
tetraethylurea	14.74	1.446	1.915	2.065	0.161
dimethylcyanamide	37.23	1.409	2.464	2.544	0.480
carbon_disulfide	2.64	1.624	10.804	10.888	0.287
dimethyl_sulfide	6.20	1.432	0.822	1.009	0.190
diethyl_sulfide	5.72	1.440	0.799	0.983	0.104
diisopropyl_sulfide	5.81	1.438	0.796	0.981	0.088
dibutyl_sulfide	4.41	1.450	0.659	0.825	0.066
tetrahydrothiophene	8.61	1.502	2.959	3.142	0.131
thiane	6.58	1.510	3.014	3.201	0.110
dimethylsulfoxide	46.45	1.477	3.394	3.460	0.634
dibutyl_sulfoxide					0.213
sulfolane	43.26	1.481	3.488	3.559	0.641

Continued on next page

Table S1 *continued*

**Medium**	ε	n	W_{3med} (mJ m^{-2})	W_{5med} (mJ m^{-2})	$\Delta G°$ (kJ mol^{-1})
thiobis2-ethanol	27.84	1.519	4.860	4.960	0.702
diethyl_sulfite	15.60	1.415	1.805	1.950	0.318
dimethyl_sulfate	50.28	1.386	2.912	2.974	0.407
diethyl_sulfate	16.20	1.414	1.843	1.986	0.344
methanesulfonic_acid		1.432			0.867
trimethylphosphate	16.39	1.395	2.032	2.174	0.513
triethylphosphate	10.79	1.403	1.511	1.683	0.318
tri-n-butylphosphate	8.91	1.422	1.206	1.388	0.146
hexamethylphosphoric_triamide	39.50	1.507	4.470	4.546	0.213
hydrogen_peroxide	70.70	1.407	2.739	2.785	1.749
hydrogen_fluoride	84	1.340	4.629	4.669	1.074
sulfuric_acid	100	1.418	2.776	2.809	0.780
ammonia	22.38	1.325	4.778	4.894	0.653
hydrazine	52.90	1.469	3.220	3.280	1.062
sulfur_dioxide	11.90	1.357	2.702	2.868	0.348
thionyl_chloride	9.25	1.516	3.701	3.881	0.635
phosphorus_oxychloride	13.90	1.484	2.775	2.929	0.463

REFERENCES

(1) Bain, C. D.; Troughton, E. B.; Tao, Y.-T.; Evall, J.; Whitesides, G. M.; Nuzzo, R. G. Formation of Monolayer Films by the Sponaneous Assembly of Organic Thiols from Solution onto Gold. *J. Am. Chem. Soc.* **1989**, *111*, 321-335.

(2) Hutt, D. A.; Leggett, G. J. Dependence of Rates of Photo-oxidation of Self-assembled Monolayers on Adsorbate Alkyl Chain Length. *J. Phys. Chem.* **1996**, *100*, 6657-6662.

(3) Hutt, D. A.; Cooper, E.; Leggett, G. J. Structure and Mechanism of Photo-oxidation of Self-assembled Monolayers of Alkylthiols on Silver studied by XPS and Static SIMS. *J. Phys. Chem. B* **1998**, *102*, 174-184.

(4) Siles-Brugge, O. *TToolbox*, https://gitlab.com/oscarsiles/TToolbox (last accessed 8/3/24).

(5) Marcus, Y. *The properties of solvents*, Wiley, 1998; pp 95–102.

(6) Lide, D. R. *CRC Handbook of Chemistry and Physics*; Taylor & Francis, 2003.
